# Exploratory study of plasma total homocysteine and its relationship to short-term outcome in acute ischaemic stroke in Nigerians

**DOI:** 10.1186/1471-2377-8-26

**Published:** 2008-07-12

**Authors:** Njideka U Okubadejo, Olajumoke O Oladipo, Adekunle A Adeyomoye, Gbolahan O Awosanya, Mustapha A Danesi

**Affiliations:** 1 Department of Medicine, College of Medicine, University of Lagos, Lagos, Nigeria; 2 Department of Chemical Pathology, College of Medicine, University of Lagos, Lagos, Nigeria; 3 Department of Radiation Biology, Radiotherapy and Radiodiagnosis, College of Medicine, University of Lagos, Lagos, Nigeria

## Abstract

**Background:**

Hyperhomocysteinemia is a potentially modifiable risk factor for stroke, and may have a negative impact on the course of ischaemic stroke. The role of hyperhomocysteinemia as it relates to stroke in Africans is still uncertain. The objective of this study was to determine the prevalence and short-term impact of hyperhomocysteinemia in Nigerians with acute ischaemic stroke. We hypothesized that Hcy levels are significantly higher than in normal controls, worsen stroke severity, and increase short-term case fatality rates following acute ischaemic stroke.

**Methods:**

The study employed both a case-control and prospective follow-up design to study hospitalized adults with first – ever acute ischaemic stroke presenting within 48 hours of onset. Clinical histories, neurological evaluation (including National Institutes of Health Stroke Scale (NIHSS) scores on admission) were documented. Total plasma Hcy was determined on fasting samples drawn from controls and stroke cases (within 24 hours of hospitalization). Outcome at 4 weeks was assessed in stroke patients using the Glasgow Outcome Scale (GOS).

**Results:**

We evaluated 155 persons (69 acute ischaemic stroke and 86 healthy controls). The mean age ± SD of the cases was 58.8 ± 9.8 years, comparable to that of controls which was 58.3 ± 9.9 years (T = 0.32; P = 0.75). The mean duration of stroke (SD) prior to hospitalization was 43.5 ± 38.8 hours, and mean admission NIHSS score was 10.1 ± 7.7. Total fasting Hcy in stroke patients was 10.2 ± 4.6 umol/L and did not differ significantly from controls (10.1 ± 3.6 umol/L; P = 0.88). Hyperhomocysteinemia, defined by plasma Hcy levels > 90^th ^percentile of controls (>14.2 umol/L in women and >14.6 umol/L in men), was present in 7 (10.1%) stroke cases and 11 (12.8%) controls (odds ratio 0.86, 95% confidence interval 0.31 – 2.39; P > 0.05). In multiple regression analysis admission NIHSS score (but not plasma Hcy) was a significant determinant of 4 week outcome measured by GOS score (P < 0.0001).

**Conclusion:**

This exploratory study found that homocysteine levels are not significantly elevated in Nigerians with acute ischaemic stroke, and admission Hcy level is not a determinant of short-term (4 week) stroke outcome.

## Background

Stroke prevention is a key component of any public health strategy aimed at reducing the rising burden of cardiovascular diseases in low and middle income countries [1]. Several risk factors for stroke have been identified and are the target of both primary and secondary preventive strategies [2-4]. It has however become increasingly apparent that apart from the traditional risk factors associated with stroke, several newer independent risk markers that are promising targets for modification, such as hyperhomocysteinemia, may contribute substantially to the risk of stroke.

Homocysteine (Hcy) is a sulphydryl-containing amino acid derived from the metabolic demethylation of the dietary essential amino acid methionine by the liver and other proliferating cells [5]. Hyperhomocysteinemia may be genetic (due to cystathione β-synthase or methylenetetrahydrofolate reductase (MTHFR) mutations) or acquired (dietary, renal impairment, drugs, and co-morbid illnesses such as leukemia) [5,6]. The evidence for an association between hyperhomocysteinemia and atherosclerotic disease has been present for over 30 years, but has only more recently gained focus [7,8]. Hyperhomocysteinemia may promote oxidative injury to the vascular bed with proliferation of vascular smooth muscle, altered endothelial function, and enhanced thrombogenicity [9-11]. Emerging evidence from epidemiologic studies supports a strong, dose-dependent, positive association between plasma Hcy level and the risk of cardiovascular diseases including stroke. Recent prospective and retrospective studies have shown that high and moderately elevated Hcy levels are potentially modifiable risk factors for stroke in all age groups, independent of the effect of smoking, cholesterol and blood pressure [12-18]. Additionally, increased levels of Hcy in blood seem to have a negative impact on the course of ischaemic stroke, significantly increasing the risk of poor recovery [19]. The role of hyperhomocysteinemia as it relates to stroke in Africans is still uncertain. The objective of this study was thus to determine the prevalence of hyperhomocysteinemia in Nigerians with acute ischaemic stroke, and the impact of hyperhomocysteinemia on short-term stroke outcome. We hypothesized that Hcy levels in acute ischaemic stroke are significantly higher than in normal persons, that Hcy levels are related to stroke severity, and hyperhomocysteinemia increases the short-term case fatality rates following ischaemic stroke in Nigerians.

## Methods

Approval of the study protocol was obtained from the Health Research and Ethics Committee of the Lagos University Teaching Hospital (LUTH), Lagos, Nigeria. The study protocol encompassed two aspects. The first was a case-control design to compare the levels of Hcy in persons on admission for acute ischaemic stroke with age- and sex-matched matched healthy controls. The second design was a prospective 4-week follow up study of the same cohort of stroke cases to determine the relationship of admission Hcy levels to short-term stroke outcome.

We recruited all consecutive first – ever acute ischaemic stroke patients presenting within 48 hours of onset of focal neurological deficit and aged > 15 years. Informed consent was obtained from all stroke cases or their proxies. All the potential participants consented to participate in the study, giving a zero non-response rate. Brain computerized tomography (CT) scans were conducted to exclude hemorrhagic stroke and other intracranial structural causes of focal neurological deficits. All CT scans were reviewed by the study consultant radiologists to confirm the diagnosis of ischaemic stroke. In a few instances where CT scan was not done, ischaemic stroke was diagnosed in cases that fulfilled both the World Health Organization [20] and Siriraj stroke score criteria [21] for ischaemic stroke. The control group comprised of healthy volunteers (including spouses of stroke cases and civil servants) matched for age (± 2 years) and gender to controls.

In each instance, the clinical history, cardiovascular and neurological examination, and evaluation of stroke status using the National Institutes of Health Stroke Scale (NIHSS) [22] were documented. Patients with NIHSS score > 13 were classified as severe stroke, while a score ≤ 13 was regarded as mild stroke. Follow-up evaluations were conducted at 4 weeks post-stroke onset. The evaluations were conducted in hospital in all instances. This was enabled by the operational unit protocol for stroke care at our institution which includes 4 week hospitalization period in all persons suffering a stroke. Outcome at 4 weeks was assessed on the Glasgow Outcome Scale [23] with a score of 1 indicating good recovery, 2 – moderate disability, 3 – severe disability, 4 – vegetative survival, and 5 – death.

### Homocysteine assay

Total plasma Hcy was determined on fasting (after 12 hours) samples drawn within 24 hours of hospitalization (within 72 hours of stroke onset in all instances, with a range of 20 – 72 hours). Control samples were also collected after a 12 hour fast. 5 ml of venous blood was collected from each subject into EDTA tubes, transported to the laboratory immediately, and separated within 1 hour of collection. Plasma was stored at minus 20°C until batched analysis after every 20 samples. Total L – Hcy was assayed by the fluorescence polarization immunoassay (FPIA) method of Schipchandler *et al *[24], using an IMx ^® ^Abbot immunoassay instrument (Abbot Laboratories, Illinois, USA). For this method, bound Hcy (oxidized form) is reduced to free Hcy which is then enzymatically converted to S – adenosyl – L – Hcy (SAH). Total free Hcy is converted to SAH by the use of SAH hydrolase, and excess adenosine SAH hydrolase converts SAH to Hcy. Excess adenosine in the pretreatment solution drives the conversion of Hcy to SAH by the bovine SAH hydrolase. A minimum volume of 50 ul of plasma is used for the assay. Mean within-run precision (n = 10) and between-run precision (n = 10) was 3.8% and 4.5% respectively for the 3 levels of controls used (low, medium and high). Abnormal total Hcy was defined as any level above the 90^th ^percentile in the distribution of Hcy in the control population.

### Statistical analysis

Data were analyzed using EPI Info ^® ^2002 and the Statistical Package for the Social Sciences (SPSS ^®^) version 13.0 for Windows^®^. The Χ^2 ^test was used to compare proportions, whilst the Student t test was used to compare the mean values of continuous variables between groups. Multiple linear regression analysis was used to assess the independent contribution of the variables in the prediction of outcome (GOS score at 4 weeks post stroke). P values < 0.05 were considered statistically significant.

## Results

The study evaluated a total of 155 persons (69 with first-ever acute ischaemic stroke – 31 women and 38 men; and 86 healthy controls – 35 women and 51 men). The mean age ± SD of the cases was 58.8 ± 9.8 years, comparable to that of controls which was 58.3 ± 9.9 years (T = 0.32; P = 0.75). There was no significant difference between the mean age ± SD of men (58.0 ± 8.9) and women (59.7 ± 11.0) with stroke (T = 0.69; P = 0.49). The clinical and demographic characteristics specific to the stroke cases, including admission Glasgow Coma Scale scores, NIHSS scores, blood pressures (systolic, diastolic and mean arterial pressures), and stroke severity (frequency of mild and severe stroke) are shown in Table 1.

**Table 1:** Demographic and clinical characteristics of stroke cases and controls

**Parameter**	**Cases**	**Controls**	
	**n = 69**	**n = 86**	**P value**
Male to female ratio	38:31	35:51	0.11
Age (years), mean ± SD	58.8 ± 9.8	58.3 ± 9.9	>0.05
Duration of stroke prior to hospitalization (hours), mean ± SD	43.5 ± 38.8	N/A	
Hypertension, n (%)	59 (85.5%)	0 (0%)	<0.00001
Diabetes mellitus, n (%)	19 (27.5%)	0 (0%)	<0.00001
Alcohol consumption, n (%)	22 (31.9%)	19 (22.1%)	0.23
Cigarette smoking, n (%)	5 (7.2%)	2 (2.3%)	0.24
Admission Glasgow coma scale score, mean ± SD	13.8 ± 2.7	N/A	
Admission NIHSS score, mean ± SD	10.1 ± 7.7	N/A	
Admission systolic blood pressure (mmHg), mean ± SD	160.6 ± 29.1	116.4 ± 29.1	<0.00001
Admission diastolic blood pressure (mmHg), mean ± SD	99.0 ± 17.9	76.9 ± 4.9	<0.00001
Admission mean arterial pressure (mmHg), mean ± SD	119.1 ± 20.8	90.0 ± 5.5	<0.00001
Stroke severity			
Mild (NIHSS ≤ 13)	53 (76.8%)		
Severe (NIHSS >13)	16 (23.2%)	N/A	

Blood pressure values for controls represent measurements at time of recruitment

### Homocysteine profile in cases compared to controls

The mean ± SD total fasting Hcy level for acute ischaemic stroke patients was 10.2 ± 4.6 umol/L. This did not differ significantly from the level in controls, which was 10.1 ± 3.6 umol/L (P = 0.88). The median Hcy for cases was 9.2 umol/L, while that of controls was 9.6 umol/L. Hyperhomocysteinemia was defined by plasma Hcy levels above the 90^th ^percentile for the control group for each gender. It was thus defined by plasma Hcy >14.2 umol/L in women and >14.6 umol/L in men. In all, 7 (10.1%) of the stroke cases and 11 (12.8%) of the controls had hyperhomocysteinemia (odds ratio 0.77, 95% confidence interval 0.27 – 2.12; P = 0.80).

### Plasma total Hcy in relation to stroke-specific characteristics and outcome

The relationship between plasma total Hcy levels and various subgroup characteristics was explored. Specifically, comparisons were made by gender, number of identified modifiable risk factors (multiple or single), presence of hypertension, diabetes mellitus, smoking status, and stroke severity on admission (Table 2).

**Table 2:** Comparison of mean plasma total Hcy (umol/L) values based on various intra-group characteristics of stroke cases overall and by gender stratification

**Variable**	**Total**	Statistics *	**Male**	**Female **		
	**N = 69**		**N(Mean ± SD)**	**N (Mean ± SD)**	**T**	**P**
Number of risk factors						
Single (n = 40)	10.3 ± 4.5	T = 0.18; P = 0.88	22 (10.0 ± 3.4)	18 (10.6 ± 5.6)	0.42	0.68
Multiple (n = 29)	10.1 ± 4.7		16 (10.7 ± 5.5)	13 (9.3 ± 3.7)	0.79	0.43
Hypertension						
Yes (n = 59)	10.1 ± 4.0	T = 0.14; P = 0.89	34 (10.7 ± 4.5)	25 (9.4 ± 3.1)	1.17	0.25
No (n = 10)	10.4 ± 7.6		4 (7.1 ± 1.4)	6 (12.5 ± 9.4)	1.10	0.29
Diabetes						
Yes (n = 19)	8.7 ± 3.3	T = 1.71; P = 0.09	6 (8.2 ± 3.1)	13 (8.9 ± 3.4)	0.44	0.66
No (n = 50)	10.7 ± 4.9		32 (10.7 ± 4.5)	18 (10.9 ± 5.6)	0.12	0.90
Smoking						
Yes (n = 5)	8.7 ± 3.4	T = 0.74; P = 0.46	5 (10.3 ± 4.7)	0	-	-
No (n = 64)	10.3 ± 4.7		33 (10.5 ± 4.5)	31 (10.0 ± 4.9)	0.41	0.68
Admission stroke severity						
Mild (NIHSS ≤ 13) (n = 53)	10.2 ± 4.9	T = 0.12; P = 0.91	32 (10.4 ± 4.4)	21 (10.0 ± 5.6)	0.29	0.78
Severe (NIHSS > 13) (n = 16)	10.1 ± 3.5		6 (9.9 4.5)	10 (10.2 3.1)	0.18	0.86

* Comparing the categories e.g. single v. multiple risk factors, hypertension present or absent, diabetes present or absent, smoking yes or no, and stroke severity mild or severe

The case fatality rate in this study was 7.2% (5/69). The degree of disability at 4 weeks post stroke in the survivors (n = 64) using the Glasgow Outcome scale as the outcome measure was as follows: Good recovery – 22 (31.9%), Moderate disability – 17 (24.6%), Severe disability – 23 (33.3%), and Vegetative – 2 (2.9%). Mean ± SD and median GOS scores at 4 weeks in cases with normal and elevated Hcy were 2.21 ± 1.07 (median = 2.0) and 3.00 ± 1.73 (median = 3.0) respectively (T = 1.73, P = 0.09).

Furthermore, using multiple linear regression analysis, we explored the possibility of a relationship between GOS score at 4 weeks (as the dependent variable) and age, admission systolic BP (SBP), diastolic BP (DBP), mean arterial pressure (MAP), admission GCS score, admission NIHSS score, and plasma Hcy as explanatory variables (Table 3). Using this model, the only variable that was significantly associated with GOS score at 4 weeks was the admission NIHSS score (P < 0.0001). Plasma Hcy level on admission was not a determinant of short term outcome as measured by the GOS score (P = 0.07).

**Table 3:** Multiple linear regression analysis of determinants of stroke outcome (GOS scores) at 4 weeks post stroke

**Variable**	**Coefficient**	**Standard error**	**P value**	**95% confidence interval**	
Age	-0.004	0.011	0.72	-0.03	0.17
Admission SBP	0.015	0.016	0.37	-0.02	0.04
Admission DBP	0.022	0.035	0.53	-0.05	0.09
Admission MAP	-0.023	0.047	0.63	-0.11	0.08
Admission GCS score	-0.063	0.054	0.25	-0.16	0.06
Admission NIHSS score	0.087	0.018	<0.0001*	0.05	0.12
Plasma homocysteine	0.041	0.023	0.07	-0.01	0.08

Dependent variable for the model is GOS score at 4 weeks. *GOS scores at 4 weeks were only significantly related to admission NIHSS scores.

## Discussion

This study was designed to prospectively explore two aspects of the relationship between fasting total plasma Hcy and acute ischaemic stroke in Nigerians. In the first instance, we sought to compare the prevalence of elevated Hcy in stroke cases compared to matched controls. Secondly, we prospectively evaluated the relationship between Hcy levels (measured within 72 hours of stroke onset) and short-term outcome in acute ischaemic stroke in Nigerians using a case-control design.

Our principal findings based on the case-control study are that the levels of plasma Hcy in cases and their age-matched controls from the same population were not significantly different, and the prevalence of hyperhomocysteinemia was similar in cases and controls. The data regarding concentrations of Hcy in stroke patients have been confounding. Lindgren *et al *compared plasma Hcy levels in the acute and convalescent periods following stroke, and found that, in contrast to several earlier studies, the concentration of plasma Hcy did not differ between cases and controls in the acute phase, and plasma Hcy levels were in fact higher in the convalescent period following stroke [25]. This finding was corroborated in a more recent study which concluded that decreased Hcy levels found on admission for acute ischaemic stroke may reflect the strength of the acute-phase response rather than a pathogenetic event [26]. Glew *et al *also evaluated Hcy levels in ischaemic stroke occurring in Northern Nigeria and found similar levels in cases and controls [27]. These findings are supported by the study of Sacco *et al *who investigated the association between various degrees of elevation of Hcy and the risk of incident ischaemic stroke in a triethnic cohort which included blacks [28]. They showed that mild to moderate elevations of total Hcy are less predictive, the vascular effects are less, and total Hcy is not a significant risk factor for ischaemic stroke among blacks. Although results of studies exploring the relationship between Hcy and ischaemic stroke have been conflicting, several other studies have reported a lack of risk of stroke attributable to hyperhomocysteinemia and have in fact on occasion shown a decline in Hcy levels in the acute post stroke period possibly representing an acute phase response [25,26,29]. It has been suggested that following an acute inflammatory reaction, serum albumin concentrations decline, and albumin being the principal plasma Hcy-binding protein, this situation may result in lowering of total plasma Hcy levels. Spence [30] has however enjoined caution in interpreting the mechanism underlying lowered Hcy levels in the immediate post-stroke period, and emphasizes the potential role of recumbency in reducing the blood levels of several analytes including Hcy. Furthermore, the Hcy-stroke association may be specific to certain pathophysiologic subtypes of stroke (such as large artery atherosclerosis), and may be masked in studies such as ours in which the vascular subtypes of ischemic stroke were not separately analyzed [15,31].

Our study also found that elevated Hcy levels were not related to the occurrence of more severe stroke. Conversely, our study results showed a weak association between severe stroke (measured by admission NIHSS scores) and lower Hcy levels. Lower Hcy levels in the acute phase of stroke may in fact represent a more severe acute phase reaction corresponding to a worse vascular insult. Perini *et al *also reported a lack of correlation between elevated plasma Hcy on admission and stroke severity [31].

In the prospective follow-up study, we found that admission hyperhomocysteinemia was not a determinant of short-term outcome (measured by the Glasgow outcome scale score at 4 weeks) following acute ischaemic stroke in our patients. The relationship between Hcy and stroke outcome remains controversial, with some studies finding an association where others have not [32,33]. If hyperhomocysteinemia occurs as an effect or epiphenomenon rather than a cause of acute ischaemic stroke as has been suggested [34], this may explain why elevated Hcy, despite not determining stroke severity at the onset, may have an effect on stroke outcome in the short term, in the same way that factors such as infections and other complications acquired in the post-acute phase can impact negatively on short-term outcome.

One of the limitations of our study is that, as is the case for studies of this nature, the pre-stroke Hcy levels of our stroke cases were not known, and so we cannot categorically determine if the Hcy levels measured represent a decline, an increase, or are reflective of pre-stroke values. An ideal study designed to address this would necessarily incorporate a cohort with pre-determined Hcy levels (e.g. at study entry), and followed up on the long-term, prospectively documenting incident strokes and eventually determining the relationship between Hcy and incident stroke, while controlling for confounders. Also, our study conclusions cannot be extrapolated to describe the relationship between Hcy and stroke outcome on the long term. We did not explore the relationship between the vascular subtype (e.g. small vessel versus large vessel disease) and total plasma Hcy levels, and cannot thus conclude on whether elevated Hcy has varying effects based on the specific vascular subtype of stroke. We also note that although blood pressure has previously been documented as an independent determinant of Hcy levels [35], and though the blood pressure measurements (systolic, diastolic, and mean arterial pressures) in our cases were significantly higher than controls, their Hcy levels were essentially similar. We acknowledge the limitations inherent in the study sample size and thus describe our study as exploratory, with the intention that further studies employing a larger sample size can be conducted in the future.

## Conclusion

This exploratory study found that homocysteine levels are not significantly elevated in Nigerians with acute ischaemic stroke, and admission Hcy level is not a determinant of short-term (4 week) stroke outcome.

## Abbreviations

Blood pressure: BP; Computerized tomography: CT; Diastolic blood pressure: DBP; Glasgow Outcome Scale: GOS; Homocysteine: Hcy; National Institutes of Health Stroke Scale: NIHSS; Standard deviation: SD; Systolic blood pressure: SBP.

## Competing interests

The authors declare that they have no competing interests.

## Authors' contributions

NUO, OOO and MAD conceptualized the study and obtained the grant for the study. NUO and MAD obtained the data. AAA and GOA obtained and reviewed all CT scans, and participated in the review of the manuscript for intellectual content. Hcy assays were conducted by OOO. NUO conducted the data analysis. NUO and OOO drafted the manuscript and reviewed its intellectual content. MAD also reviewed the manuscript for intellectual content.

## Pre-publication history

The pre-publication history for this paper can be accessed here:


